# Directed evolution of an m^6^A eraser for site-selective epitranscriptome editing

**DOI:** 10.21203/rs.3.rs-7958216/v1

**Published:** 2025-11-04

**Authors:** Chuan-Hui Wang, Huiqing Zhou

**Affiliations:** 1. Department of Chemistry, Merkert Chemistry Center, Boston College, Chestnut Hill, MA 02467, USA

## Abstract

*N*^6^-methyladenosine (m^6^A) is a critical RNA modification, but tools to manipulate it in cells remain limited. Current m^6^A demethylases (FTO and ALKBH5) exhibit low catalytic efficiency, hindering their applications in epitranscriptomic research and biotechnology. Here, we develop a fluorescence-based directed evolution platform that enables rapid selection of m^6^A demethylases with enhanced activity. Using this platform, we generated FTO-818, an evolved FTO variant with 13-fold increased catalytic efficiency against m^6^A compared to wild-type FTO. FTO-818 demonstrates superior demethylation performance both on purified biological RNAs *in vitro* and on endogenous m^6^A when overexpressed in human cells. Furthermore, we constructed a targeted m^6^A editor by fusing FTO-818 to catalytically-dead Cas13b (dCas13b-FTO-818), achieving efficient, site-specific removal of m^6^A from individual mRNA and lncRNA. This directed evolution platform and the resulting FTO-818 enzyme provide powerful new tools for investigating m^6^A biology and advancing applications in epitranscriptomic research and biotechnology.

## INTRODUCTION

*N*^6^-methyladenosine (m^6^A) is a prevalent modification in the eukaryotic transcriptomes^1–4^ and regulates multiple aspects of RNA metabolism and function, such as stability^5–8^, splicing^9,10^, translation^11,12^, nuclear export^13^, and transcription^14^. Increasing evidence has revealed the critical relevance of m^6^A in physiology and diseases. For example, effector proteins of m^6^A (*i.e*., writers, erasers, and readers) are frequently dysregulated in multiple human cancers^15–19^. However, dysregulation of an effector protein is often accompanied by concurrent changes of hundreds to thousands of m^6^A sites^20,21^. It remains challenging to pinpoint the functional output caused by a m^6^A site(s) on a specific transcript(s). Tools that allow direct modulation of m^6^A in living cells are critical for investigating the functions of a specific m^6^A site. CRISPR-based systems have emerged as promising tools to install or remove m^6^A on specific transcripts, where an m^6^A eraser or writer enzyme was fused with a catalytically deactivated Cas-family protein (dCas) with a guide RNA directing the eraser or writer to an m^6^A site of interest^22–25^. However, a critical limitation is that all available tools utilize the wild-type writers or erasers for m^6^A, which have low catalytic efficiency^22,26–28^. Notably, the known human m^6^A eraser wild-type FTO (wtFTO) or ALKBH5 demethylates m^6^A with 970–1617 fold reduced catalytic efficiency (*k*_cat_/*K*_m_ = 0.06 or 0.1 min^−1^μM^−1^ for wtFTO or ALKBH5)^28,29^, compared to a homologous bacterial enzyme AlkB demethylating m^1^A (*k*_cat_/*K*_m_ = 97 min^−1^μM^−1^) in RNA oligonucleotides^30^. The low catalytic efficiency hinders the application of m^6^A erasers for epitranscriptome research and biotechnological applications such as promoting root growth in plants^31^.

Enhancing the catalytic efficiency of m^6^A erasers is key to advancing next-generation m^6^A editing tools. Yet, there have not been methods available for rapidly evolving m^6^A erasers with improved catalytic activity. Reported targeted mutagenesis of wtFTO increased the binding affinity of wtFTO to the nucleic acid substrate by up to 16-fold but did not alter demethylation activity significantly^32^. Directed evolution provides a powerful strategy for rapidly evolving protein variants with desired properties for processing RNA modifications^33–35^. Here, we report the development of a fluorescence-based directed evolution platform to rapidly evolve m^6^A demethylase with improved catalytic efficiency. We establish the platform using the *N*^6^-methyldeoxyadenosine (6mA)-modified Lettuce aptamer as a substrate and the wtFTO as the target protein to evolve. Lettuce aptamer is fluorogenic upon binding with a fluorophore^36,37^ and incorporation of 6mA at the fluorophore binding site abolishes the fluorogenic property. The fluorescence is activated when the 6mA gets demethylated by wtFTO or an FTO variant. Using the fluorescence-based readout for demethylation, we performed two rounds of directed evolution screening 1,272 FTO variants. The directed evolution resulted in six FTO variants with improved demethylation activities compared to the wtFTO, of which the variant FTO-818 shows the highest activity. We demonstrate that FTO-818 shows enhanced demethylation performance of m^6^A in biological RNAs *in vitro* and site-specific m^6^A editing in human cells.

## RESULTS

### Lettuce aptamer-based fluorescence activation assay for detecting demethylation of 6mA.

**Figure 1.**
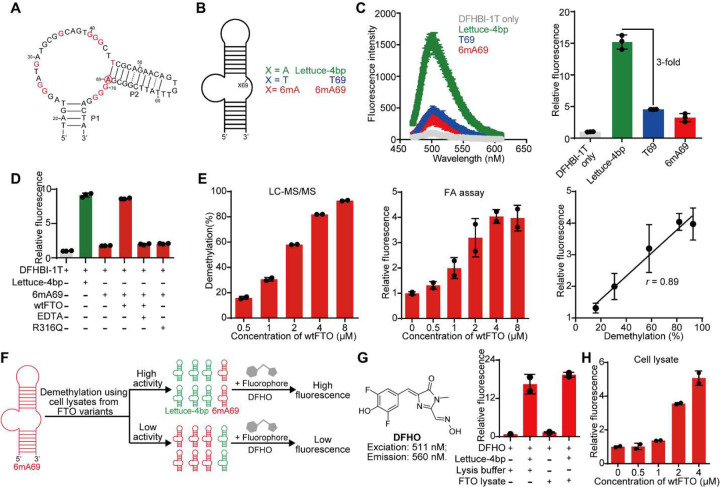
Development of the fluorescence activation (FA) assay for detecting demethylation and directed evolution of FTO. (**A**) Predicted secondary structure of Lettuce-4bp. Mutation-intolerant residues are colored in red, and dA69 is circled. (**B**) Schematic representation of three aptamers: Lettuce-4bp, T69, and 6mA69. (**C**) Shown on the left are fluorescence emission spectra of Lettuce-4bp, T69, and 6mA69 with DFHBI-1T (excitation at 455 nm). 2.5 μM of aptamer was incubated with 1.25 μM of DFHBI-1T in the DNA binding buffer at room temperature for 1 hour, before the reading was taken. Fluorescence intensities at 505 nm are plotted for each aptamer on the right. All intensities are normalized to that from DFHBI-1T-only. Data are presented as mean ± SD with n=3 biological replicates. (**D**) Demethylation activity of wtFTO was measured by the FA assay with conditions listed below. Relative fluorescence (in reference to the DFHBI-1T alone assay) was plotted for each demethylation condition. 2.5 μM of aptamer was incubated with 4 μM wtFTO or the R316Q FTO mutant at 37 °C for 2 hours. 5 mM EDTA was added if present. Data are presented as mean ± SD with n=3 biological replicates. (**E**) Shown are the demethylation of 6mA69 quantified by LC-MS/MS demethylation assay (left) and FA assay (middle) across varying enzyme concentrations. The calibration curve (right) shows the correlation between fluorescence intensities by FA assay and demethylation percentage by LC-MS/MS. Data are presented as mean ± SD with n=2 biological replicates. (**F**) Schematic illustration of the directed evolution platform for wtFTO by screening FTO variants in the crude cell lysate form via FA assay using 6mA69 as the substrate. (**G**) Fluorescence intensities of Lettuce-4bp with DFHO in the presence or absence of crude cell lysate from BL21 cells. Chemical structure of DFHO is shown on the left. Fluorescence intensities at 560 nm of Lettuce-4bp with DFHO (excited at 511nm) are plotted against listed conditions. Data are presented as mean ± SD with n=2 biological replicates. (**H**) FA assay of 6mA69 treated with crude cell lysates containing varying amounts of purified wtFTO. Fluorescence intensity at 560 nm was normalized to the signal from the cell lysate–only group. Data are presented as mean ± SD with n=2 biological replicates.

Prior work from Jaffrey reported fluorescence activation upon demethylation of the m^6^A-substituted RNA aptamer Broccoli^38^; however, RNA aptamers are prone to degradation during protein directed evolution efforts, where variants are often screened without purification. As wtFTO removes *N*^6^-methylation on both single-stranded RNA and DNA^26^, we choose to establish a fluorescence-based reporter for demethylation with fluorogenic DNA aptamer. Lettuce is a fluorogenic DNA aptamer upon binding with fluorophores (*e.g*., DFHBI-1T, DFHO) that mimic the chromophore of the green fluorescence protein^36,37^. We work with a truncated version of Lettuce “Lettuce-4bp” with 4 bp in the P1 stem (Figure 1A), as the truncated aptamer shows comparable fluorescence compared to the full-length one^36^, and provides ease for introducing chemical modification during synthesis. Twelve residues on Lettuce-4bp are crucial for the fluorogenic property and intolerant for mutations and only one of twelve residues is a deoxyadenosine (dA): dA69 (Figure 1A)^36^. Interestingly, the N6-H6 of dA69, or the equivalent dA43 in the high-resolution crystal structure of Lettuce construct (PDB ID: 8FHX)^37^, forms a direct hydrogen-bonding interaction with O6 of dG26, which stacks with the bound DFHBI-1T (**Supplementary Figure 1A**). We hypothesized that introducing *N*^6^-methylation at dA69 can reduce the fluorogenic property of the Lettuce-4bp aptamer. We designed three synthetic DNA aptamers: the Lettuce-4bp as reported^36^, the “T69” aptamer with dA69 mutated to dT69, and the “6mA69” aptamer with dA69 modified to be 6mA69 (Figure 1B, **Supplementary Table 1**, and Methods). Consistent with the previous report^36^, Lettuce-4bp exhibits maximal fluorescence at 505 nm in the presence of DFHBI-1T (Figure 1C); the T69 aptamer shows a 3-fold reduction in fluorescence intensity at 505 nm, demonstrating the intolerance of the A69 site to mutations. Interestingly, the 6mA69 aptamer shows a similar reduction of fluorescence intensity as T69, compared to Lettuce-4bp (Figure 1C). We attribute the decreased fluorescence of T69 and 6mA69 aptamers to the significantly weakened binding between the aptamer and DFHBI-1T (**Supplementary Figure 1B**).

Next, we assessed whether we can apply the 6mA69 aptamer as a fluorescence activation reporter for 6mA demethylase’s activity. We prepared human FTO protein with an *N*-terminal His tag (*i.e*., wtFTO) via recombinant expression from *E.coli* BL21 (DE3) cells (Methods and **Supplementary Figure 2**). We set up the fluorescence activation (FA) assay by incubating the DNA aptamer (Lettuce-4bp or 6mA69) with wtFTO and cofactors (Fe^2+^, and 2-oxoglutarate or 2OG) for demethylation, annealing the product DNA aptamer in the presence of DFHBI-1T, and recording fluorescence (Methods). Interestingly, 6mA69 shows 4.8-fold increased fluorescence upon demethylation by wtFTO, compared to the reaction without wtFTO. The fluorescence intensity generated by demethylated 6mA69 aptamer is comparable with fluorescence from the Lettuce-4bp (Figure 1D). Meanwhile, we performed control FA assays for 6mA69 aptamer treated with deactivated FTO by adding an excess amount of EDTA^39^, which chelates an essential demethylation cofactor Fe^2+^, or replacing the wtFTO with the catalytically-dead R316Q FTO mutant^26^ (Methods and **Supplementary Figure 3**). Results show that both deactivated FTO and R316Q FTO mutant show abolished fluorescence activation, which confirms that the enhanced fluorescence from the 6mA69 aptamer treated by wtFTO is due to the demethylation of 6mA69 (Figure 1D).

Next, we investigated whether the FA assay could semi-quantitatively report the extent of demethylation (*i.e*., the demethylation percentage of 6mA-modified DNA). To achieve variable extents of demethylation, we performed the demethylation reaction of the 6mA69 aptamer with gradually diluted concentrations of wtFTO, with a constant reaction time^40^. Meanwhile, we quantified the extent of demethylation for each reaction using the orthogonal LC-MS/MS method as a benchmark^26^ (Methods). The results show that the 6mA69 aptamer treated by wtFTO ranging from 0.5 μM to 8 μM resulted in demethylation percentages from 16% to 93% (Figure 1E and **Supplementary Figure 4**). Meanwhile, we performed the same series of demethylation reactions by FA assays; the resulted fluorescence intensities correlate positively (Pearson’s *r* = 0.89) with the demethylation percentages determined by LC-MS/MS (Figure 1E). This suggests that the FA assay can be semi-quantitative for reporting the extent of demethylation of the 6mA69 aptamer.

### Fluorescence activation assay with crude cell lysate.

For directed evolution, we reason that any demethylase variant that exhibits higher catalytic efficiency for demethylating 6mA would generate enhanced fluorescence, measured by the FA assay, compared to the wild-type demethylase (Figure 1F). We focus on evolving wtFTO due to its higher *in vitro* catalytic activity than the other known m^6^A eraser ALKBH5^41^. To enable rapid screening of FTO variants, we tested whether we could perform the FA assay using FTO in the crude cell lysate form instead of purified protein. DFHBI-1T appears not to be appropriate for this purpose, as we encountered high background fluorescence at 505 nm (maximum emission for DFHBI-1T) from the crude cell lysate from BL21 cells alone, even without any Lettuce-4bp aptamer or DFHBI-1T added (**Supplementary Figure 5A**). To address the interference of fluorescence from the crude cell lysate, we adopted a different fluorophore DFHO with red-shifted excitation/emission wavelengths when binding with Lettuce (511/560 nm)^36^, to replace DFHBI-1T (455/505 nm) in the FA assay (Figure 1G). Notably, we did not observe background fluorescence from cell lysates at the wavelengths for DFHO (Figure 1G). We further validated that DFHO is suitable for the FA assay using the purified wtFTO protein, producing semi-quantitative fluorescence upon demethylation (**Supplementary Figures 5B, 5C**).

Next, we performed the FA assay by treating 6mA69 with the crude cell lysate with overexpressed wtFTO (Methods). However, we did not observe fluorescence activation for the wtFTO lysate-treated aptamer relative to the control (*i.e*., lysate without overexpressed FTO) (**Supplementary Figure 5D**). With this observation, we assessed whether the presence of complex components in the crude cell lysate inhibits the activity of wtFTO. We titrated purified wtFTO protein into cell lysates (non-induced). Encouragingly, we observed fluorescence activation at 1 μM or higher concentration of titrated wtFTO in the crude cell lysate (Figure 1H). This suggests that the activity of wtFTO is not completely inhibited by other components in the crude cell lysate. When there could be an FTO variant with enhanced demethylation activity in the lysate form during directed evolution, we could capture that through fluorescence activation.

### Directed evolution of wtFTO.

**Figure 2.**
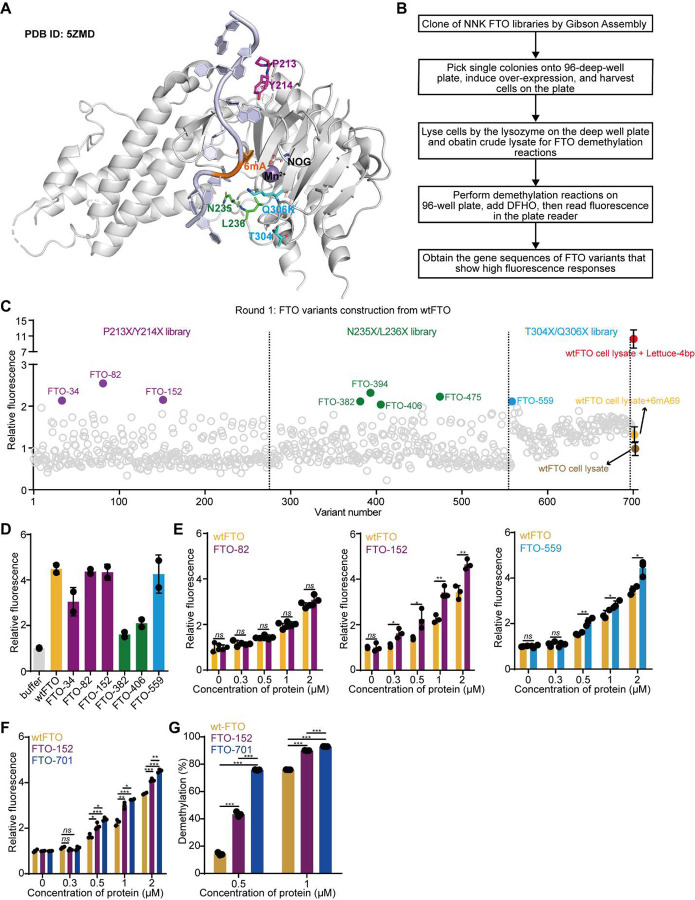
Directed evolution of wtFTO (round 1). (**A**) Shown is the crystal structure of wtFTO in complex with 6mA-ssDNA (TCT6mATATCG) and cofactors Mn^2+^ and NOG (PDB ID: 5ZMD). The 6mA is colored in orange. The six amino acids targeted for mutagenesis during the first round of directed evolution are color-coded by individual protein libraries: P213/Y214 in purple, N235/L236 in green, and T304/Q306 in blue. (**B**) Shown is the flow chart of the directed evolution procedure. (**C**) Shown are fluorescence intensities measured by the FA assay for all FTO variants screened during the first round of directed evolution. Fluorescence intensity at 560 nm for each variant was normalized to the intensity from the wtFTO cell lysate. The control FA assays during the screening are color-coded with the positive controls in red, negative controls in brown, and baseline controls in yellow. Data for the control assays are shown as mean ± SD (n = 10 biological replicates). Variants exhibiting elevated fluorescence above the baseline are highlighted in color corresponding to individual library as noted in panel **A**. (**D**) Shown are FA assay results with 4 μM purified proteins. Fluorescence data are normalized to the non-protein control “buffer”. Data are presented as mean ± SD with n=2 biological replicates. (**E**) Shown are fluorescence intensities of 2.5 μM 6mA69 treated by FTO-82, FTO-152, FTO-559, and wtFTO at 37 °C for 2 hours, across a series of low protein concentrations by the FA assay. Data are presented as mean ± SD with n=3 biological replicates. The two-tailed t-test was used to assess the statistical significance of the difference between two samples, with the *p*-value indicated as “*ns*” for *p* ≥ 0.05, “*” for *p* < 0.05, “**” for *p* < 0.01, or “***” for *p* < 0.001. This same statistical method was used to compare differences between samples in the current study. (**F**) Shown are FA assay results for FTO-152, FTO-701, and wtFTO across a range of low protein concentrations. Data are presented as mean ± SD with n=3 biological replicates. (**G**) Demethylation percentage of 2.5 μM 6mA69 treated by 0.5 or 1 μM purified wtFTO, FTO-152, and FTO-701 quantified by LC-MS/MS demethylation assay. Data are presented as mean ± SD with n=3 biological replicates.

To initiate the directed evolution campaign, we chose to permutate amino acids at or near the interface between the single-stranded DNA (ssDNA) substrate and FTO based on the crystal structure of the recognition complex (PDB ID: 5ZMD), inspired by the reported mutagenesis study where mutations at the interface improved substrate affinity for wtFTO^32,41^. We constructed three initial libraries for wtFTO by permutating two amino acids in each library: P213X/Y214X, N235X/L236X, and T304X/Q306X, where X indicates any one of the twenty natural amino acids (Figure 2A). Among these sites, P213, Y214, N235, and L236 are interacting with the backbone of ssDNA through electrostatic interactions. Though T304 and Q306 are located slightly far from the interface, the side chain of K306 in the Q86K/Q306K double mutant of FTO can interact with the ssDNA backbone shown by the crystal structure (Figure 2A)^32^.

To prepare the FTO libraries, we introduced permutation at each amino acid site using NNK codon, where N is for A/T/C/G and K is G/T. Libraries were cloned and transformed into BL21 (Figure 2B). A single colony represents a unique genotype, which results in a unique variant of wtFTO. We picked several colonies and validated the diversity of the genotypes at targeted codon positions by Sanger Sequencing (**Supplementary Figure 6**). To perform the directed evolution, we prepared the crude cell lysates of FTO variants on a 96-deep well plate following the published protocol^33^ (Methods). We then performed the demethylation of 6mA69 using crude cell lysates containing FTO variants and then added DFHO to record fluorescence signals across the plate (Figure 2B). For each plate, we express wtFTO in six wells to perform duplicated positive controls (with Lettuce-4bp), negative controls (no aptamer), accounting for the highest and lowest fluorescence of the FA assay, as well as baseline controls (with 6mA69) serving as references for the activity of wtFTO in the lysate form.

By screening 279, 279, and 141 FTO variants for the P213X/Y214X, N235X/L236X, and T304X/Q306X libraries, respectively, we identified multiple variants that show increased fluorescence compared to wtFTO (Figure 2C). We picked the top eight variants (with at least one variant from each library) that show promising fluorescence signals (colored in Figure 2C) and performed reproducibility test. Six out of the eight selected variants (FTO-34, FTO-82, FTO-152, FTO-382, FTO-406, and FTO-559) showed significantly improved fluorescence response compared to the wtFTO lysate, tested by three biological replicates with cell lysates (**Supplementary Figure 7A**). We further characterized the genotypes of the six variants (**Supplementary Figure 7B**) and proceeded with testing their activities with purified enzymes (Methods and **Supplementary Figures 8A**, **8B**).

We subjected these purified FTO variants to the FA assay with a high enzyme concentration at 4 μM to check their activities. At this concentration, the purified wtFTO can remove over 80% of the *N*^6^-methylation from 6mA69 (Figure 1E). The results show that three variants FTO-82, FTO-152, and FTO-559 produce comparable fluorescence signal as the wtFTO (Figure 2D). However, the other three variants (FTO-34, FTO-382, and FTO-406) show lower fluorescence than wtFTO. We ruled out the possibility that the reduced signals for these variants arose from any DNase contamination in the purified proteins (**Supplementary Figure 8C**). FTO-34, FTO-382, and FTO-406 turned out less active than the wtFTO despite that they were selected from the lysate screening, and we did not pursue further tests of these three variants (Figure 2D).

To compare demethylation efficiencies of FTO-82, FTO-152, and FTO-559 with wtFTO, we performed the FA assay with gradually diluted enzymes. Interestingly, FTO-152 (P213R/Y214K) and FTO-559 (T304S/Q306T) show significantly higher fluorescence compared to wtFTO under diluted enzyme concentration(s) (Figure 2E), suggesting that these two variants could have improved activities. We then cloned and purified another FTO variant FTO-700 (P213R/Y214K/T304S/Q306T) that combines all four mutations (**Supplementary Figure 8D**). However, FTO-700 did not show increased demethylation compared to wtFTO tested by both FA assay and LC-MS/MS (**Supplementary Figures 8D, 8E**). As Jia *et al* show that Q306K mutation increases its binding affinity to 6mA-modified ssDNA by 10-fold^32^, we examined whether a combinatorial variant of FTO-152 and Q306K will further improve the demethylation performance (**Supplementary Figure 8F**). Excitingly, FTO-701 (P213R/Y214K/Q306K) exhibited further improved demethylation activity compared to wtFTO and FTO-152 based on the FA assay (Figure 2F) and the LC-MS/MS demethylation assay (Figure 2G and **Supplementary Figure 8G**). Results confirmed significantly improved demethylation percentages for the FTO variants (FTO-701 > FTO-152 > wtFTO) against 6mA69 (Figure 2G), suggesting a synergistic effect of the combined mutations.

**Figure 3.**
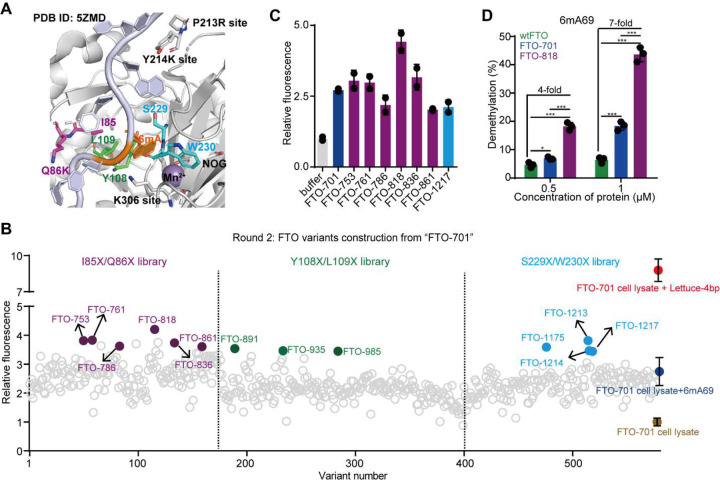
Directed evolution of wtFTO (round 2). (**A**) Zoomed-in view of the active site of wtFTO (PDB ID: 5ZMD). Six residues targeted for mutagenesis in the second round are color-coded by individual protein libraries: I85/Q86 in purple, Y108/L109 in green, and S229/W230 in blue. Amino acid sites that are mutated in the FTO-701 genotype (residues 213, 214, and 306) are labeled on the structure. (**B**) Shown are fluorescence intensities measured by the FA assay for all FTO variants screened during the second round of directed evolution. Fluorescence intensity at 560 nm for each variant was normalized to the intensity from the FTO-701 cell lysate. The control FA assays during the screening are color-coded with the positive controls in red, negative controls in brown, and baseline controls in dark blue. Data for the control assays are shown as mean ± SD (n = 23 biological replicates). Variants exhibiting elevated fluorescence above the baseline are highlighted in color corresponding to individual libraries as noted in panel **A**. (**C**) Shown are demethylation activities of FTO variants from the second round measured by FA assay using 2 μM purified proteins and 20 minutes for the demethylation reaction. Fluorescence intensities are normalized to that of the non-protein control “buffer”. Data are presented as mean ± SD with n=2 biological replicates. (**D**) Shown are demethylation percentages of 2.5 μM 6mA69 treated by 0.5 or 1 μM purified wtFTO, FTO-701, and FTO-818, measured by the LC-MS/MS demethylation assay. Data are presented as mean ± SD with n=3 biological replicates.

As FTO-701 is the most promising variant so far, we performed the second round of directed evolution based on FTO-701, by introducing mutations at amino acid positions in proximity to the 6mA and metal binding sites^32^ (Figure 3A). We prepared three FTO variant libraries: I85X/Q86X, Y108X/L109X, and S229X/W230X (**Supplementary Figure 9**), and all variants are generated based on the genotype of FTO-701. Considering the improved activity of FTO-701, we shortened the demethylation reaction time from 2 hours (in the first round) to 20 minutes to allow more stringent selection. We identified 6, 3, and 4 variants that show above-baseline fluorescence levels from I85X/Q86X, Y108X/L109X, and S229X/W230X libraries, respectively (colored in Figure 3B). Reproducibility tests show seven variants (six from I85X/Q86X and one from S229X/W230X) exhibit significantly elevated fluorescence responses compared to FTO-701 (**Supplementary Figure 10A**) and represent distinct genotypes (**Supplementary Figure 10B**). To rigorously compare their relative activities, we purified the seven variants (**Supplementary Figures 11A, 11B**) and performed the FA assay with 2 μM purified FTO variants and a 20-minute demethylation reaction time (Figure 3C). Excitingly, we found that FTO-818 showed markedly higher fluorescence activation than all other tested variants, including FTO-701. Three other variants from the I85X/Q86X library (FTO-753, FTO-761, and FTO-836) also showed improved activities compared to FTO-701 (Figure 3C and **Supplementary Figure 11C**). Interestingly, in these four selected variants, the Q86 mutates into a more hydrophobic residue (A, V, Y, or I) and the 85 position remains as a hydrophobic residue (L, C, or F) in the mutants (**Supplementary Figure 10B**), highlighting benefits for 6mA recognition through favoring the hydrophobic effect at residues 85 and 86. Data from the LC-MS/MS demethylation assay for wtFTO, FTO-701, and FTO-818 demethylating 6mA69 further validated the activities observed by the FA assay, which showed that FTO-818 presents a 4–7 fold increased demethylation percentage compared to wtFTO (Figure 3D). Therefore, we conclude FTO-818 (I85C/Q86Y/P213R/Y214K/Q306K) as the most promising FTO variant for further characterizations and applications.

### FTO-818 shows enhanced demethylation for m^6^A in RNA oligonucleotides.

**Figure 4.**
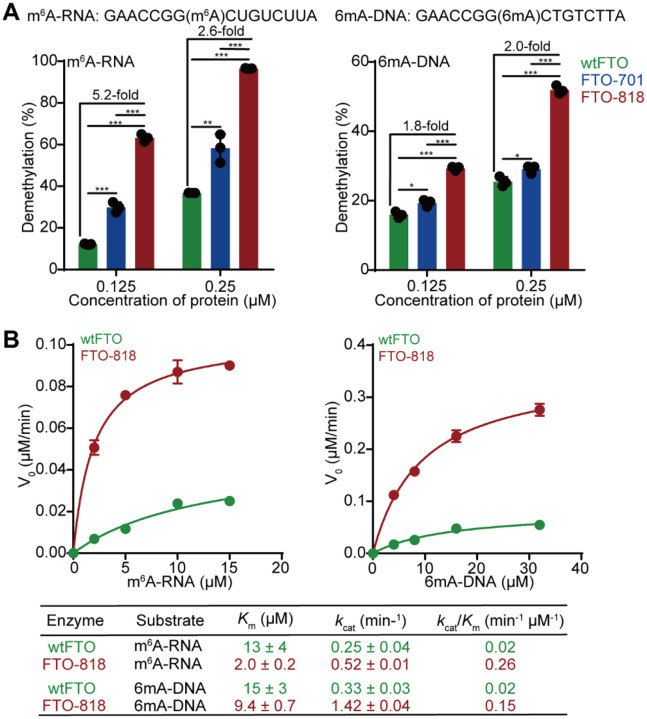
Characterization of catalytic activities of FTO-818 against m^6^A in RNA oligonucleotide. (**A**) Shown are demethylation percentages of 1 μM m^6^A-RNA (left) and 6mA-DNA (right) by 0.125 or 0.25 μM purified wtFTO, FTO-701, and FTO-818 quantified by the LC-MS/MS demethylation assay. Data are presented as mean ± SD with n=3 biological replicates. (**B**) Shown are Michaelis-Menten kinetics measured for wtFTO and FTO-818 demethylating m^6^A-RNA and 6mA-DNA. Data are presented as mean ± SD with n=2 or 3 biological replicates. Fitted *K*_m_ and *k*_cat_ values are shown in the table.

As FTO variants were evolved against 6mA in DNA, we assessed the demethylation activities for wtFTO, FTO-701, and FTO-818 against a m^6^A-modified RNA oligonucleotide (m^6^A-RNA) via the LC-MS/MS demethylation assay (**Supplementary Table 1**). We first benchmarked the demethylation activity of our purified wtFTO under previously reported conditions: treating 10 μM m^6^A-RNA with 0.5 μM purified wtFTO yielded 80% demethylation of the substrate, comparable to the ~75% reported previously^32^ (**Supplementary Figure 12A**). Increasing the wtFTO concentration to 1 μM in the demethylation reaction resulted in complete substrate demethylation (**Supplementary Figure 12A**). Next, we performed the LC-MS/MS demethylation assay under diluted enzyme concentrations (0.125 μM or 0.25 μM) to provide sufficient dynamic range to evaluate potentially enhanced FTO variants (**Supplementary Figure 12B** and Methods). FTO-818 exhibited the highest demethylation percentage among the three tested proteins (wtFTO, FTO-701, and FT-O818), with a 5.2-fold increase in the demethylation percentage compared to wtFTO at 0.125 μM enzyme concentration (Figure 4A and **Supplementary Figure 12C**). Meanwhile, the other three variants (FTO-753, FTO-761, and FTO-836), selected in the second round of directed evolution, show slightly improved demethylation activity against m^6^A-RNA relative to FTO-701, though not as prominent as FTO-818 (**Supplementary Figure 12C**).

To compare the demethylation activities of FTO variants between RNA versus DNA substrates, we prepared a modified DNA oligonucleotide “6mA-DNA” that carries the same sequence as “m^6^A-RNA” (Figure 4A). We performed the LC-MS/MS demethylation assay with the three FTO proteins: wtFTO, FTO-701, and FTO-818, under the same assay conditions as that used for m^6^A-RNA. The results show the demethylation activities as FTO-818 > FTO-701 > wtFTO for 6mA-DNA, presenting the same trend as 6mA69 as the substrate where 6mA locates in a different DNA sequence context (Figure 4A, Figure 3D, and **Supplementary Figure 12D**). Notably, FTO-818 demethylates more m^6^A-RNA than 6mA-DNA under the same conditions, suggesting m^6^A-RNA could be a better substrate for FTO-818 than 6mA-DNA. It was observed before that m^3^U in RNA is a better substrate for wtFTO than m^3^T in DNA of the same sequence^42^. Yet, broader sequence contexts must be tested for evaluating general substrate preference of FTO-818 for DNA versus RNA.

To gain deeper insights into the catalytic properties of FTO-818, we measured Michaelis-Menten kinetics for FTO-818 and wtFTO against m^6^A-RNA or 6mA-DNA (Methods). wtFTO presents the catalytic efficiency (*k*_cat_/*K*_m_) = 0.02 min^−1^μM^−1^ for m^6^A in m^6^A-RNA, where m^6^A locates in a GGm^6^ACU sequence context (Figure 4B and **Supplementary Figure 13A**). This measured efficiency is comparable to the previously reported value (*k*_cat_/*K*_m_ = 0.06 min^−1^μM^−1^ for m^6^A in a 5-mer RNA oligonucleotide)^28^. wtFTO catalyzes the demethylation reactions in DNA and RNA with comparable kinetics (Figure 4B and **Supplementary Figure 13C**). FTO-818 shows 7.5-fold or 13-fold higher catalytic efficiency than wtFTO in demethylating 6mA-DNA or m^6^A-RNA, respectively (Figure 4B and **Supplementary Figures 13B, 13D**), consistent with the previously observed promoted demethylation activity for FTO-818 compared to wtFTO (Figure 4A). Interestingly, the kinetics results show that the enhanced catalytic efficiencies for FTO-818 may arise from distinct mechanisms for DNA versus RNA: for m^6^A-RNA, we observe a substantially increased *K*_m_ for FTO-818 compared to wtFTO, whereas for 6mA-DNA substrate, FTO-818 shows primarily enhancement in *k*_cat_ (Figure 4B). It is possible that the five amino acid substitutions in FTO-818, spanning in the binding interface and catalytic vicinity, could differentiate the catalytic mechanisms for RNA versus DNA.

### FTO-818 shows enhanced demethylation efficiency against m^6^A, m^6,6^A, and m^1^A in biological RNAs over wtFTO.

As FTO-818 shows a marked enhancement in catalytic activity against m^6^A in RNA oligonucleotides, we continue testing the demethylation performance and substrate selectivity of FTO-818 against m^6^A and other modifications in physiologically relevant RNAs via the LC-MS/MS demethylation assay. Here, we focused on adenosine modifications m^6^A and m^1^A that were known to be substrates for endogenous wtFTO^43^. In the meantime, we assessed m^6,6^A, which was previously reported as a substrate for ALKBH5 *in vitro*^44^ but not yet characterized for FTO, as well as Am as a negative control of which the 2’-O-methylation is not a substrate for wtFTO^28^.

**Figure 5.**
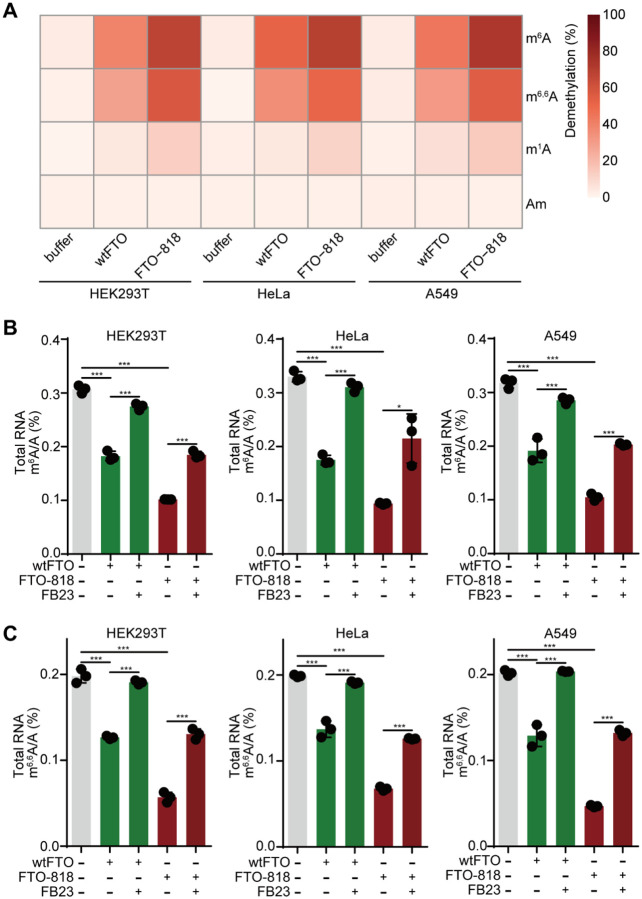
Assessment of catalytic activity and substrate selectivity of FTO-818 in biological RNAs. (**A**) Shown is a heatmap summarizing the demethylation percentage of modifications m^6^A, m^6,6^A, m^1^A, and Am in biological RNAs by FTO-818 and wtFTO, measured by the LC-MS/MS demethylation assay. Biological RNAs purified from three human cell lines: HEK293T, HeLa, and A549 are labeled. (**B**-**C**) Inhibition of FTO-818 and wtFTO by the inhibitor **FB23** for demethylating m^6^A and m^6,6^A in total RNAs *in vitro*. Shown are LC-MS/MS quantifications of modification abundance of m^6^A (**B**) and m^6,6^A (**C**) in total RNAs from each cell line treated by wtFTO or FTO-818, in the presence or absence of **FB23**. Data are presented as mean ± SD with n=3 biological replicates.

We extracted total RNA and purified polyA-enriched RNA or tRNA from multiple human cell lines including HEK293T, HeLa, and A549 cells, and quantified m^6^A in polyA-enriched RNA, m^1^A in tRNA, m^6,6^A, and Am from fragmented total RNA, before and after FTO treatments via LC-MS/MS (Methods and **Supplementary Figure 14A**). FTO-818 consistently achieves higher demethylation percentage for m^6^A in polyA-enriched RNAs from all three cell lines, compared to wtFTO, demonstrating its superior demethylation activity in the biological RNA contexts *in vitro* (Figure 5A and **Supplementary Figure 14B**). Interestingly, both wtFTO and FTO-818 show demethylation activity against m^6,6^A from fragmented total RNA samples, in which FTO-818 presents a higher demethylation percentage than wtFTO, similar in trend as m^6^A (Figure 5A and **Supplementary Figure 14C**). Under our demethylation assay conditions, wtFTO showed negligible demethylation on m^1^A from tRNA *in vitro*, whereas FTO-818 showed a slight improvement in the extent of demethylation of m^1^A (Figure 5A and **Supplementary Figure 14D**). As expected, neither wtFTO nor FTO-818 removes 2’-O-methylation in Am (Figure 5A and **Supplementary Figure 14C**). Together, these findings highlight the promoted demethylation activity of m^6^A in biological RNAs of FTO-818. FTO-818 overall shows a similar substrate selectivity profile as wtFTO across the four tested RNA methylations. Both enzymes demethylate m^6^A and m^6,6^A effectively, while they present much reduced activity for m^1^A and no activity for Am.

To investigate the potential molecular mechanisms that lead to the promoted activity of FTO-818 over wtFTO, we characterized the secondary structures and thermal stabilities of the two proteins. wtFTO and FTO-818 produce identical profiles measured by the circular dichroism (CD) spectroscopy (**Supplementary Figure 15A**) and same thermal stabilities measured by the thermal shift assay (**Supplementary Figure 15B**). These results suggest no major changes in overall secondary structures and thermostabilities for FTO-818, compared to wtFTO. The molecular mechanisms for the promoted activity can be localized to the substrate and catalytic cofactor binding sites. To further compare the substrate binding modes between wtFTO and FTO-818, we examined if both enzymes can be inhibited by a known selective wtFTO inhibitor **FB23**^45^. **FB23** inhibits FTO activity upon binding to the same pocket as the modified nucleobase substrates, with an IC_50_ = 0.06 μM^45^. We performed the demethylation reaction for 0.5 μM wtFTO or FTO-818 in the presence of excess amount of **FB23** (5 μM), where we expect to observe near complete inhibition of wtFTO activity. As expected, demethylation activity of wtFTO is almost completely abolished for both m^6^A and m^6,6^A; interestingly, the demethylation activity of FTO-818 was only partially inhibited by 5 μM **FB23** (Figures 5B, 5C, and **Supplementary Figure 15C**). This is consistent with the markedly decreased *K*_m_ values for FTO-818 compared to wtFTO (Figure 4B), suggesting increased substrate affinity. Meanwhile, we cannot rule out the possibility that there can be structural changes in the binding pocket of the modified base in FTO-818 that disfavor binding of **FB23**.

### FTO-818 shows improved demethylation of m^6^A than wtFTO in living cells.

**Figure 6.**
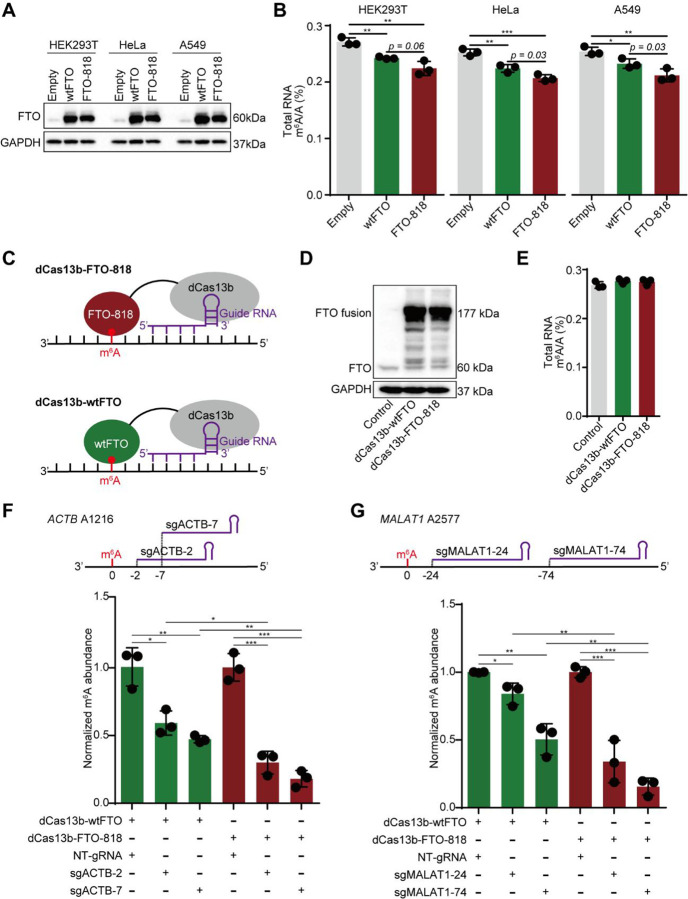
Promoted activity of FTO-818 over wtFTO in living cells. (**A**) Verification of FTO overexpression in HEK293T, HeLa, and A549 cells by Western blot. Anti-FTO antibody was used for western blotting of FTO, and GAPDH is shown as a control. (**B**) Shown are LC-MS/MS quantifications of m^6^A abundance in total RNAs extracted from HEK293T, HeLa, and A549 cells with overexpression of wtFTO or FTO-818. Data are presented as mean ± SD with n=3 biological replicates. (**C**) Schematic representations of dCas-FTO fusion constructs for site-specific m^6^A editing. (**D**) Shown is western blotting of dCas13b-wtFTO and dCas13b-FTO-818 proteins over-expressed in HEK293T cells using an anti-FTO antibody. Western blotting of GAPDH is shown as a control. (**E**) Quantification of m^6^A abundance in total RNAs extracted from HEK293T cells over-expressing dCas13b-wtFTO or dCas13b-FTO-818. (**F**) Shown above is the targeted m^6^A1216 site in *ACTB* mRNA and the relative positions of two gRNAs (sg*ACTB*-2 and sg*ACTB*-7). Shown below is the normalized m^6^A abundance at *ACTB* A1216 measured by SELECT following co-transfection of dCas13b-wtFTO or dCas13b-FTO-818 with the corresponding NT-gRNA or on-target gRNAs. Data are presented as mean ± SD with n=3 biological replicates. (**G**) Shown above is the targeted m^6^A2577 site in *MALAT1* lncRNA and the relative positions of two gRNAs (sg*MALAT1*-24 and sg*MALAT1*-74). Shown below is the normalized m^6^A abundance at *MALAT1* A2577 measured by SELECT following co-transfection of dCas13b-wtFTO or dCas13b-FTO-818 with the corresponding NT-gRNA or on-target gRNAs. Data are presented as mean ± SD with n=3 biological replicates.

Encouraged by the enhanced demethylation activity of FTO-818 *in vitro*, we proceeded to evaluate the activity of FTO-818 in living cells. First, we over-expressed FTO-818 or wtFTO upon transient transfection in HEK293T, HeLa, and A549 cells (**Supplementary Figures 16A, 16B**). An empty vector was also transfected as a negative control. We performed RT-qPCR and western blotting to confirm the over-expression of wtFTO or FTO-818 relative to the control, on the mRNA and protein levels, respectively (Figure 6A and **Supplementary Figure 16C**). We then quantified m^6^A in total RNAs extracted from transfected cells using LC-MS/MS. The results reveal a significant decrease in m^6^A levels from endogenous RNAs when wtFTO is over-expressed, compared to the control cells. Moreover, the m^6^A level gets further reduced when FTO-818 is over-expressed, suggesting FTO-818 catalyzes a greater degree of global demethylation when over-expressed in living cells, compared to wtFTO, across all three tested cell lines (Figure 6B and **Supplementary Figure 16D**). In contrast, neither over-expression of wtFTO nor FTO-818 alters the endogenous level of m^6,6^A, which suggests m^6,6^A may not be a substrate for FTO in cells (**Supplementary Figure 16D**) despite that FTO can demethylate m^6,6^A *in vitro* (Figure 5A).

Given that FTO-818 shows greater activity than wtFTO in cells, we reason that it would be promising to deploy FTO-818 for site-specific removal of m^6^A in a specific transcript (Figure 6C). We constructed dCas13b-FTO-818 by fusing the catalytically inactivated type VI-B Cas13 enzyme from *Prevotella* sp. *P5–125* (dPspCas13b)^46^ to the *N*-terminal side of FTO-818 (“dCas13b-FTO-818”), with an NLS sequence and FLAG tag at the *C*-terminal end of the construct (**Supplementary Figure 17A**). The design of dCas13b-FTO-818 was based on the previously reported and readily available dCas13b-wtFTO construct^47^. Both constructs were transiently transfected into the HEK293T cells (Methods). We confirmed the expression of the dCas13b-FTO fusion proteins (~177 kDa) via western blot with an anti-FTO antibody (Figure 6D). Notably, transfection with dCas13b-fusion constructs did not alter the endogenous expression levels of endogenous FTO (~60 kDa) (Figure 6D) and overall endogenous m^6^A levels (Figure 6E and **Supplementary Figure 17B**).

Next, we assessed the site-specific m^6^A editing efficiency of dCas13b-FTO-818 and dCas13b-wtFTO, focusing on two well-characterized m^6^A sites in HEK293T cells: m^6^A1216 in *ACTB* and m^6^A2577 in *MALAT1*^48,49^. We prepared two guide RNAs (gRNAs) for each site following the reported gRNA sequences^49^. To target one m^6^A site, we transfected the HEK293T cells with the dCas fusion construct together with the gRNA vector. As a negative control for m^6^A editing, a non-targeting gRNA (*i.e*., NT-gRNA) vector was transfected instead of an on-target gRNA (Methods). Following the cell treatment, the m^6^A levels at a single site are quantified based on the reported SELECT method^50^.

m^6^A1216 in *ACTB* shows a 41–53% decrease in m^6^A levels when targeted by the dCas13b-wtFTO and the two on-target gRNAs (sg*ACTB*-2 and sg*ACTB*-7), relative to the NT-gRNA control. The two gRNA sequences show comparable site-specific demethylation efficiency (Figure 6F). Excitingly, cells treated by dCas13b-FTO-818 and the same gRNAs exhibit 70–82% reduction in m^6^A levels. The site-specific m^6^A demethylation efficiency of dCas13b-FTO-818 is significantly greater than the dCas13b-wtFTO construct with biological replicates, for both on-target gRNAs (Figure 6F).

Similarly, we prepared two gRNA constructs (sg*MALAT1*-24 or sg*MALAT1*-74) to target the m^6^A2577 in the long non-coding RNA *MALAT1*. For the dCas13b-wtFTO construct, the sg*MALAT1*-24 gRNA only yields 16% removal of m^6^A2577 while sg*MALAT1*-74 shows 50% m^6^A removal (Figure 6G). In this case, the m^6^A editing efficiency shows a strong gRNA dependence, which reveals the importance of gRNA optimization efforts for each site of targeting with the wtFTO. Strikingly, with the evolved FTO-818, the editing efficiency of m^6^A2577 by dCas13b-FTO-818 reaches 66–84% for both gRNAs, achieving substantial removal of m^6^A with either gRNA (Figure 6G).

To assess potential off-target activity of the dCas13b-fusion proteins, we examined m^6^A levels at non-targeted sites. Specifically, we quantified the m^6^A level of m^6^A2577 on *MALAT1* in cells transfected by the dCas-FTO proteins and gRNAs for *ACTB*, where both sg*ACTB*-2 and sg*ACTB*-7 act as off-target gRNAs for m^6^A2577 on *MALAT1*. For both dCas13b-wtFTO and dCas13b-FTO-818 constructs, there are no changes in the m^6^A levels for m^6^A 2577 on *MALAT1*, suggesting that m^6^A2577 is not hit by off-target gRNAs (**Supplementary Figure 17C**). Vice versa, we analyzed m^6^A1216 in *ACTB* in cells transfected with gRNAs targeting *MALAT1* and observed no off-target editing (**Supplementary Figure 17D**). Together, these results demonstrate that dCas13b-FTO-818 significantly enhances site-specific m^6^A demethylation in cells, enabling the development of the next-generation site-specific m^6^A removing tools, which can hopefully achieve effective site-specific editing with a broad tolerance of gRNA design and provide the potential to perform transcriptome-wide m^6^A editing.

## DISCUSSION

In this study, we developed a fluorescence-based directed evolution platform for m^6^A demethylases, demonstrated by evolving the wtFTO. Beyond FTO, the directed evolution platform can be adapted into systems for evolving other modification effector proteins from humans or other organisms. Meanwhile, 6mA69 aptamer offers a convenient fluorescence turn-on reporter for demethylation *in vitro*; this property can be repurposed into a fluorescence-based high-throughput screening assay for discovering or evaluating small molecule modulators targeting the epitranscriptome. During our directed evolution efforts, we recognize that the fluorescence signal generated by the crude cell lysates expressing FTO variants is typically low relative to the background, which renders the likelihood of identifying false-positive variants such as FTO-34, FTO-382, and FTO-406 (Figure 2D). Moreover, lysate-based screening can be affected by factors such as protein expression levels or nonspecific interactions of complex components in the cell lysate with the 6mA69 aptamer, leading to both false-negative and false-positive identifications. Therefore, in our current workflow, it is crucial to test the reproducibility of fluorescence activation with biological replicates of the promising variants after screening (**Supplementary Figures 7A and 10A**). It would be helpful to develop fluorogenic DNA aptamers with enhanced binding affinity to the fluorophore and brightness, to increase fluorescence signal in cell lysate, or enable potential cell-based selection if the DNA aptamer is stable and strongly fluoresces in cells.

The directed evolution of wtFTO yielded the evolved FTO-818 that shows markedly improved catalytic efficiency for m^6^A. As wtFTO was frequently utilized in transcriptome-wide mapping methods for detecting m^6^A in mammalian cells, such as m^6^A-REF-seq^51^ and m^6^A-SAC-seq^52^, by creating a pool of RNAs with m^6^A removed as a control for m^6^A identification. The development of FTO-818 provides a more efficient enzyme to further advance these approaches. It would be interesting to examine if catalysis by FTO-818 would go through the same catalytic steps as the wtFTO or not, *e.g*., whether FTO-818 could produce same intermediates during catalysis such as the hm^6^A^53^. If so, FTO-818 can be utilized in the metabolic labeling strategies such as m^6^A-SEAL-seq^53^, for endogenous m^6^A sites.

Excitingly, FTO-818 exhibits superior m^6^A demethylation in human cells, providing a powerful tool for effective editing of m^6^A modifications, which sheds light on future development of m^6^A editing tools, such as multiplexed m^6^A editing and even CRISPR screening of endogenous m^6^A sites via designed gRNA libraries, to allow rapid interrogation of functions of explicit m^6^A site(s). We acknowledge that transcriptome-wide analyses of m^6^A editing are necessary to comprehensively evaluate potential off-target effects of dCas13b-FTO-818, and to derive whether we can target multiple sites with a uniform gRNA design strategy. To further modulate the target specificity and adaptability of dCas-FTO-818, fusion of FTO-818 with other Cas family proteins such as dCasRx^48^ and R-IscB^54^ could lead to other promising editing tools.

## METHODS

### Sample Preparations

#### DNA and RNA oligonucleotides.

DNA oligonucleotides (Lettuce-4bp, T69, 6mA69, and 6mA-DNA) and RNA oligonucleotide (m^6^A-RNA) (**Supplementary Table 1**) were synthesized by Integrated DNA Technologies (IDT) and dissolved in water (Fisher Scientific, BP561–1) for usage in *in vitro assays*.

#### Plasmids.

The pET28a-wtFTO plasmid was a gift from the He lab at the University of Chicago. The R316Q plasmid was generated by replacing the arginine residue at position 316 in wtFTO with glutamine using Gibson Assembly. The FTO variant plasmids, including P213X/Y214X, N235X/L236X, and T304X/Q306X, were constructed by introducing NNK codons at the corresponding positions in the wtFTO sequence. FTO-700 plasmid was constructed from FTO-152 plasmid by introducing T304S and Q306T mutations, whereas FTO-701 plasmid was constructed by introducing the Q306K mutation into FTO-152 plasmid, both through Gibson Assembly. The FTO variant plasmids, including I85X/Q86X, Y108X/L109X, and S229X/W230X, were constructed by introducing NNK codons at the corresponding positions in the FTO-701 plasmid. Individual FTO variant plasmids were subsequently obtained from these libraries as single clones carrying specific amino acid substitutions generated by NNK mutagenesis. Human wtFTO and FTO-818 coding sequences were cloned into the pcDNA expression vector using Gibson Assembly to construct pcDNA-wtFTO and pcDNA-FTO-818 plasmids. The dCas13b-wtFTO plasmid (Addgene, 223696) and the dCas13b non-targeting gRNA plasmid (Addgene, 223698) were purchased from Addgene. We constructed the plasmid for expressing the dCas13b-FTO-818 construct by replacing the human gene sequence encoding wtFTO in the dCas13b-wtFTO plasmid with the one encoding FTO-818 using Gibson Assembly. Plasmids expressing gRNAs for *ACTB* and *MALAT1* were cloned by replacing the non-targeting gRNA sequence in the dCas13b non-targeting gRNA plasmid with the on-target sequences (**Supplementary Table 1**) using Gibson Assembly. A summary of plasmids used in this study is provided in **Supplementary Table 2**.

#### Commercial enzymes and antibodies.

Nuclease P1 (NEB, B0660S), FastAP Thermosensitive Alkaline Phosphatase (Thermo Fisher Scientific, EF0654), BST DNA polymerase (NEB, M0537S), SplintR ligase (NEB, M0375S), and DNase I (Fisher Scientific, FEREN0521) were obtained from the indicated suppliers. Antibodies against FTO, GAPDH, and HRP-conjugated anti-rabbit secondary antibody were purchased from Cell Signaling Technology (31687T, 2118T, 7074P2).

#### Protein expression and purification.

Recombinant human FTO truncated at the *N*-terminal 31 residues (FTO_ΔN31_) was overexpressed in *E.coli* BL21 (DE3) and purified as previously described with minor modifications^55^. Briefly, a 5 mL overnight culture was used to inoculate 1 L of LB medium containing 30 μg/mL kanamycin and grown at 37 °C for about 3 hours until the OD_600_ reached 0.6–0.8. Protein expression was induced with 0.5 mM isopropyl β-D-1-thiogalactopyranoside (IPTG), followed by incubation at 16 °C for 16 hours. Cells were harvested and lysed by sonication in 20 mL lysis buffer (20 mM Tris-HCl, pH 8.0, 300 mM NaCl, 1 mM dithiothreitol (DTT)) containing protease inhibitors (Thermo Fisher Scientific, A32953). The lysate was clarified by centrifugation at 11,000 rpm for 15 minutes at 4 °C. Genomic DNA was removed by adding 1% (w/v) streptomycin sulfate, followed by a second centrifugation. The supernatant was loaded onto His60 Ni Superflow Resin column (TaKaRa, 635660), and proteins were eluted with a 10–500 mM imidazole gradient in the lysis buffer. Eluted fractions were collected in 2 mL tubes and analyzed by 12% SDS-PAGE. Fractions containing predominantly the targeted protein were pooled together and concentrated to around 3 mL using a concentrator (Sigma-Aldrich). The concentrated protein was then desalted and buffer-exchanged into storage buffer (50 mM Tris-HCl, pH 7.5, 150 mM NaCl, 5% glycerol, 0.04% Triton X-100, and 1 mM DTT) using a PD-10 desalting column (GE Healthcare). The desalted proteins were concentrated to ~10 mg/mL, aliquoted, flash-frozen by liquid nitrogen, and stored at −80 °C. Protein concentrations were measured via absorbance at 280 nm, with 1 absorbance unit approximated to 1 mg/mL protein using a Nanodrop spectrophotometer (Thermo Fisher Scientific). FTO variants were overexpressed and purified using the same methods.

#### Small molecules.

DFHBI-1T (HY-110251), DFHO (HY-136277), and FB23 (HY-137187) were ordered from MedChemExpress. Nucleosides: guanosine (G) was ordered from Alfa Aesar (A11328), *N*^6^-methyladenosine (m^6^A) was ordered from AmBeed (A170736), *N*^6^*,N*^6^-dimethyladenosine (m^6,6^A) was ordered from MedChemExpress (HY-101984), and *N*^1^-methyladenosine (m^1^A) was ordered from Cayman Chemical (16937). Deoxynucleosides: 2’-deoxyguanosine (dG) and *N*^6^-methyl-2’-deoxyadenosine (6mA) were ordered from Fisher Scientific (AAJ6074103 and AAJ64961MD).

#### Biochemical Assays.

##### MS analysis of intact proteins.

Purified wtFTO and FTO variants were analyzed using an Agilent 1260 Infinity HPLC coupled with the 6230 ESI time-of-flight (ESI-TOF) mass spectrometer (MS). Protein samples were injected onto a C8 column (100 × 4.5 mm Phenomenex Aeris 3.6-μM Widepore XB-C8) and separated with an increasing gradient of 5–95% buffer B (5% water, 95% acetonitrile, and 0.1% formic acid) in buffer A (95% water, 5% acetonitrile, and 0.1% formic acid). Eluted proteins were injected into MS and analyzed in positive-ion mode. Total protein masses were calculated by deconvolution using MagTran (Amgen).

##### Fluorescence emission spectroscopy.

Fluorescence measurements were performed following previously reported methods with minor modifications^36,37^. Briefly, DNA aptamers (**Supplementary Table 1**) were heated at 75 °C for 5 minutes and immediately cooled on ice for 5 minutes. DNA aptamers solutions (5 μM, 20 μL) were mixed with an equal volume of DNA binding buffer (40 mM HEPES, pH 7.5, 100 mM KCl, and 1 mM MgCl_2_) containing 2.5 μM DFHB-1T or DFHO. After incubation at room temperature for 1 hour in the dark, 40 μL of each reaction was transferred to a black polypropylene 384-well round-bottom plate (Cellvis) and fluorescence was measured on a Synergy^™^ Neo2 Multimode Microplate Reader (BioTek). The excitation wavelengths were set to 455 ± 10 nm for DFHBI-1T and 511 ± 10 nm for DFHO, respectively. The ranges of emission wavelengths were recorded within 470–610 nm for DFHBI-1T and 532–700 nm for DFHO, respectively. Background fluorescence was determined using DNA-free controls containing 1.25 μM DFHBI-1T or DFHO in the same binding buffer.

##### Fluorescence activation (FA) assay with purified protein.

FA assays were performed in two steps, including demethylation and fluorescence emission spectroscopy in 50 μL total volume per reaction. Firstly, 2.5 μM 6mA69 was treated by wtFTO or its variants in the demethylation buffer containing 40 mM HEPES (pH 7.5), 100 mM KCl, and 1 mM MgCl_2_, 280 μM (NH_4_)_2_Fe(SO_4_)_2_, 2 mM L-ascorbic acid, 300 μM 2-oxoglutarate (2OG), and 1 mM DTT. Note that (NH_4_)_2_Fe(SO_4_)_2_, L-ascorbic acid, and 2OG are freshly dissolved from solid and prepared in the buffer right before performing the FA assay. wtFTO or its variants were then added with a specific concentration (0.3–8 μM) as noted with the presented data. The demethylation reactions were prepared by adding the buffer components first, followed by adding FTO proteins, and then the 6mA69 aptamer. The demethylation reaction was incubated at 37 °C for the indicated times (2 hours or 20 minutes) and terminated by heating at 75 °C for 10 minutes. Next, we performed fluorescence emission spectroscopy measurement. We prepared stock solutions for DFHBI-1T or DFHO at 10 mM in DMSO. We added DFHBI-1T or DFHO to the demethylation reaction mixture, with a final concentration of 1.25 μM. The sample was annealed by heating at 70 °C for 5 minutes, then slowly cooling down to room temperature for 1 hour in the dark. Fluorescence emission was measured using a microplate reader as described. To inhibit the demethylation activity of wtFTO, 5 mM EDTA was added to the reaction mixture prior to the addition of wtFTO protein. To assess potential DNase contamination, 2.5 μM Lettuce-4bp was used in place of 6mA69 in parallel reactions.

##### LC-MS/MS demethylation assay for oligonucleotides.

Demethylation reactions were performed under the same conditions as described in the FA assay with purified proteins. 2.5 μM 6mA69 (Figures 1E, 2G, and 3D), 1 μM m^6^A-RNA (Figure 4A, **Supplementary Figures 12B, 12C**),10 μM m^6^A-RNA (**Supplementary Figure 12A**) or 1 μM 6mA-DNA (Figure 4A) was used as substrate. Reactions were incubated at 37 °C for the indicated times and heat-inactivated at 75 °C for 10 minutes. Samples were then subjected to OCC clean-up according to the manufacturer’s instructions (ZYMO RESEARCH, D4061) and eluted in 50 μL RNase-free water. Eluted DNA/RNA was digested into nucleosides by adding 1 μL nuclease P1 in 10 μL Nuclease P1 reaction buffer, followed by incubation at 37 °C for 30 minutes and heat-inactivated at 75 °C for 10 minutes. Subsequently, 1 μL alkaline phosphatase in 10 μL FastAP buffer was added, and the mixture was incubated at 37 °C for 30 minutes and heat-inactivated at 75 °C for 10 minutes. Nucleoside mixtures were analyzed by LC-MS/MS using an Agilent 1200 series HPLC coupled to an Agilent 6460 Triple Quadrupole. Separation was performed on an Accucore RP-MS column (50 mm × 2.1 mm, 2.6 μm) with a gradient of buffer A (0.1% formic acid in water) and buffer B (0.1% formic acid in acetonitrile). The mass spectrometer was operated in positive MRM mode with the following parent-to-product ion transitions (m/z): 266.13 → 150.1 for 6mA, 268.11 → 152.1 for dG, 282.12 → 150.0 for m^6^A, and 284.1 → 152.0 for G.

##### Preparation of crude cell lysate for FA assay.

The pET28a-wtFTO plasmid was transformed into *E.coli* BL21 (DE3). Single colonies of wtFTO were picked onto 50 mL tubes containing 5 mL LB medium supplemented with 30 μg/mL kanamycin and incubated overnight at 37 °C with shaking. 100 μL of overnight culture was transferred into 1 mL of fresh LB medium containing 30 μg/mL kanamycin in 12 mL Falcon round-bottom tubes. Cultures were grown at 37 °C for 3 hours, followed by incubation at 16 °C for 16 hours. Cells were lysed directly in the tubes by adding 80 μL lysis buffer (20 mM Tris-HCl, pH 7.5, 300 mM NaCl, and 1 mg/mL lysozyme). Suspensions were vortexed until no visible pellets remained, incubated at 16 °C with shaking for 1 hour, and centrifuged at 4,000 rpm for 30 minutes at 4 °C. 40 μL of clarified supernatant was collected from each tube. To access the background signal from FTO-expression cell lysates, FA assays were performed under the same conditions used for purified protein, except that 5 μL crude cell lysate or lysis buffer was added in place of purified protein. Either 2.5 μM Lettuce-4bp or 6mA69 was used as the substrate.

#### Directed Evolution.

##### Cloning and preparation of crude lysate for FTO variant libraries.

FTO variant libraries were constructed using Gibson assembly with primers containing NNK at the targeted mutation sites. Single colonies from each NNK library were picked onto 96-deep-well plates containing 1 mL LB medium and 30 μg/mL kanamycin, which were incubated overnight at 37 °C with shaking. On each 96-deep-well plate, we picked 6 colonies of wtFTO or FTO-701 to express control proteins during the first and second rounds of directed evolution, respectively. 100 μL of each overnight culture was transferred into 1 mL of fresh LB medium with 30 μg/mL kanamycin in 96-deep-well plates. The rest of the overnight culture was stored at −80 °C for subsequent genotyping of FTO variants. The diluted cell cultures were grown at 37 °C for 3 hours (OD_600_ ~ 0.6–0.8 typically), when protein expression was induced with 0.5 mM IPTG, followed by incubation at 16 °C for 16 hours. Cells were harvested by centrifugation at 4,000 rpm for 30 minutes at 4 °C. The cell pellets were lysed directly in the deep-well plates by adding 80 μL lysis buffer (20 mM Tris-HCl, pH 7.5, 300 mM NaCl, and 1 mg/mL lysozyme) to each well. The suspensions were vortexed until no visible pellets remained, incubated at 16 °C with shaking for 1 hour, and centrifuged at 4,000 rpm for 30 minutes at 4 °C. 40 μL of supernatant was collected in the 96-well PCR plate (Fisher Scientific) for performing FA assay.

##### FA assay for screening FTO variants.

FA assays were performed under the same conditions as those described for the purified protein assay, except that 50 μL reaction mixtures containing 5 μL crude lysates of individual FTO variants or control FTO were used. To increase the stringency of selection, the demethylation reaction time was shortened to 20 minutes in the FA assay screening for the second round of directed evolution. After performing FA assay in 96-well PCR plate, 40 μL of each reaction product was transferred into a black polypropylene 384-well round-bottom plate for fluorescence emission spectroscopy measurement.

##### Reproducibility test of lysate-based FA assay and genotyping of FTO variants.

The assay was performed under the same conditions as previously described for screening FTO variants, except that frozen FTO variant stocks stored at −80 °C were inoculated into 1 mL of LB medium with 30 μg/mL kanamycin in 14 mL round-bottom Falcon tubes and incubated overnight at 37 °C with shaking. Subsequently, 100 μL of each overnight culture was transferred into 1 mL of fresh LB medium with 30 μg/mL kanamycin in the same types of tubes. And the reproducibility test, plasmids from FTO variants showing enhanced fluorescence were extracted and analyzed by Sanger sequencing (GENEWIZ).

##### Michaelis-Menten Kinetics.

To determine the kinetic parameters of wtFTO and FTO-818, demethylation assays were performed using either synthetic m^6^A-RNA or 6mA-DNA as substrates (**Supplemental Table 1**). Each reaction (total volume = 50μL) was prepared in 40 mM HEPES buffer (pH 7.5), containing 100 mM KCl, 1 mM MgCl_2_, and 1 mM DTT. Cofactor, including 280 μM (NH_4_)_2_Fe(SO_4_)_2_, 2 mM L-ascorbic acid, and 300 μM 2OG, were first added to the buffer, followed by the addition of wtFTO or FTO-818 (200 nM for m^6^A-RNA or 250 nM for 6mA-DNA). Reactions were initiated by adding varying concentrations of substrates (2, 5, 10, or 15 μM m^6^A-RNA and 4, 8, 16, or 32 μM 6mA-DNA) and incubated at 37 °C for the indicated times. Reactions were quenched by heating at 75 °C for 10 minutes. The resulting DNA or RNA samples were purified by OCC and digested to single nucleosides using nuclease P1 and alkaline phosphatase, and the products were analyzed by LC-MS/MS as described above. For each reaction time point, the depletion of m^6^A and 6mA was calculated by quantifying the total amount of modification in the sample and subtracting the remaining amount at each time point from the initial amount measured at 0 minute. For each substrate concentration, initial reaction rates (V_0_) were obtained as the slope of the fitted linear line when plotting the depletion of m^6^A (or 6mA) at each time point against time. Reaction velocities were plotted against substrate concentrations, and kinetic parameters (*K*_m_ and *k*_cat_) were determined by fitting the data to the Michaelis–Menten equation using GraphPad Prism 6.

##### Cell culture of human cell lines.

The HEK293T, HeLa, and A549 cell lines were generously provided as gifts from the Weerapana lab and Johnson lab at Boston College, which were originally obtained from ATCC. Cells were cultured in DMEM medium (Gibco) supplemented with 10% fetal bovine serum (FBS; Corning) and 1x penicillin/streptomycin (P/S; Gibco) on 15cm dishes, and incubated at 37 °C with 5% CO_2_. When reaching 90%−100% confluency, cells were lifted with trypsin-0.25% EDTA (Gibco) and passaged at a ratio of 1:4.

##### RNA extraction and purification from human cell lines.

Biological replicates of the extracted RNAs for subsequent modification analysis come from cultured cells from different passages. Below, we describe protocols for RNA extraction and purification for each biological replicate. ~200 μg total RNA was extracted from HEK293T, HeLa, or A549 cells using TRIzol reagent (Invitrogen) according to the manufacturer’s instructions. Residual genomic DNA was removed by treating 70 μg of total RNA with DNase I in 1× reaction buffer containing MgCl_2_ at 37 °C for 30 minutes. The reaction was terminated by adding 15 μL of 50 mM EDTA, followed by heat inactivation at 65 °C for 10 minutes. RNA was then recovered by ethanol precipitation. After DNase treatment, three parallel RNA preparations were performed: (i). Total RNA fragmentation: 20 μg of DNase I-treated total RNA was fragmented using RNA fragmentation buffer (NEB) and incubated in a preheated thermal cycler at 94 °C for 5 minutes. The reaction was stopped by adding 5 μL of 10× fragmentation stop solution, followed by purification using the RNA Clean & Concentrator kit (Zymo Research, D4061). (ii). Poly(A)^+^ mRNA enrichment: 400 ng of Polyadenylated mRNA was enriched from ~30 μg of DNase I-treated total RNA using Dynabeads Oligo(dT)25 (Invitrogen, 61005) according to the manufacturer’s protocol. (iii). tRNA isolation: 100 ng of tRNA was isolated from DNase I-treated total RNA using denaturing PAGE purification following previously reported protocols^39^.

##### LC-MS/MS demethylation assay for biological RNAs.

The purified RNA samples from HEK293T, HeLa, and A549 cells were subjected to *in vitro* demethylation reactions: 400 ng of purified mRNA, 1,500 ng of total RNA, or 100 ng of tRNA from each cell line was incubated with 0.5 μM wtFTO or FTO-818 in the demethylation buffer with a total of 50 μL of reaction volume at 37 °C for 1 hour. Treated RNAs were purified by OCC and digested into mononucleosides as described above. Nucleoside quantification was performed by LC-MS/MS: m^6^A was quantified in Poly(A)^+^ mRNAs; m^1^A was quantified in tRNAs; m^6,6^A and A_m_ were quantified in fragmented total RNAs. The following multiple reaction monitoring (MRM) transitions were used: 284→152 for G, 282→150 for m^6^A, 296→164 for m^6,6^A, 282→136 for A_m_, and 282→150 for m^1^A.

##### Inhibition of FTO demethylation by FB23.

To probe the catalytic mechanisms of wtFTO and FTO-818 towards m^6^A or m^6,6^A by small molecules, we applied the reported wtFTO inhibitor **FB23**^45^ during the demethylation reaction of fragmented total RNA and monitored the changes in m^6^A or m^6,6^A levels by LC-MS/MS. The **FB23** powder was first dissolved in DMSO, making a 20mM stock solution. 1.5 μg of fragmented total RNA was treated by 0.5 μM of wtFTO or FTO-818 in the demethylation buffer with 5 μM of **FB23**. To perform this demethylation, **FB23** was pre-incubated with FTO protein at room temperature for 10 minutes in the demethylation buffer before the addition of RNA substrate. As a negative control, equal volume of DMSO was added replacing **FB23**.

##### Circular dichroism (CD) spectroscopy.

CD spectroscopy was performed to analyze the secondary structures of wtFTO and the FTO-818 variant. Measurements were conducted on a JASCO J-1100 spectropolarimeter using a 1 mm path length cuvette. Purified proteins were prepared at 20 μM in 20 mM Tris-HCl (pH 7.5), 150 mM NaCl. Spectra were recorded from 180 nm to 260 nm, with each final spectrum representing the average of four consecutive scans to enhance the signal-to-noise ratio. Baseline correction was carried out by subtracting the spectrum of the buffer alone from each protein spectrum.

##### Thermal shift assay.

Thermal shift assays were performed to assess the thermostability of wtFTO and the FTO-818 variant, following a previously reported protocol with slight modifications^55,56^. Each 25 μL reaction contained 4 μM recombinant protein and 5× SYPRO Orange dye (Invitrogen). Samples were heated from 25 °C to 75 °C at 0.02 °C/s^−1^ using a QuantStudio^™^ 3 Real-Time PCR System (Thermo Fisher Scientific). Fluorescence was monitored at 0.02 °C intervals with excitation at 470 nm and emission at 570 nm. Raw fluorescence data were normalized by setting the minimum and maximum fluorescence values of each run to 0% and 100%, respectively. Melting temperatures (*T*m) were determined by nonlinear fitting of the normalized data to the Boltzmann Sigmoid equation using GraphPad Prism 6.0.

##### Over-expression of wtFTO and FTO-818 in human cells.

HEK293T, HeLa, or A549 cells were seeded in a 6-well plate and cultured in DMEM medium with 10% FBS (without P/S) overnight. Upon reaching 70–90% confluency, transient transfections were performed using Lipofectamine 3000 (Thermo Fisher Scientific, L3000001) following the manufacturer’s instructions. Briefly, 5 μg of plasmid DNA (pcDNA-Empty, pcDNA-wtFTO, or pcDNA-FTO-818) was mixed with 3.75 μL of Lipofectamine 3000 in 250 μL of Opti-MEM medium (Gibco) and incubated at room temperature for 10–15 minutes to allow complex formation. The DNA-lipid complexes were then added to the cells. Cells were harvested 48 hours post-transfection for downstream analyses.

##### RNA expression quantification by reverse transcription-quantitative PCR (RT-qPCR).

Total RNA was extracted from cells using TRIzol reagent (Invitrogen) following the manufacturer’s protocol. For each sample, 250 ng total RNA was then reverse transcribed into cDNA using the PrimeScript RT reagent Kit (Takara Bio) protocol in a 10 μL RT reaction. Subsequently, 4 μL of RT reaction was used as the template in the total 20 μL qPCR reaction containing 1x Universal SYBR Green Fast qPCR Mix (ABclonal) and 0.2 μM gene-specific forward and reverse primers (**Supplementary Table 1**). qPCR reactions were performed in the QuantStudio^™^ 3 Real-Time PCR System. The expression level of a target gene was reported in reference to the *β-actin* expression in the same sample by calculating 2^−Δ*Ct*^, where ΔCt = Ct_*FTO*_ - Ct_*β-actin*_. The relative expression of a gene in an overexpression wtFTO or FTO-818 cell sample was reported by normalizing the gene expression level against the expression of the same gene in the control sample (e.g. pcDNA-Empty).

##### Western blotting.

Transfected cells were washed three times with PBS and lysed on ice for 20–30 minutes in RIPA lysis buffer (Thermo Fisher Scientific, A32955) containing protease inhibitors, prepared by adding one tablet per 10 mL of RIPA lysis buffer according to the manufacturer’s instructions. The supernatant of cell lysates was collected by centrifugation at 12,000 rpm for 20 minutes at 4 °C and denatured at 99 °C for 10 minutes with SDS loading buffer. Total protein concentrations were quantified by Coomassie (Bradford) protein assay kit (Thermo Fisher Scientific, 23200), and ~5 μg of total protein per sample was loaded onto 12% SDS-PAGE gels. Gels were run at 120V for ~75 minutes and subsequently transferred to a 0.45 μM PVDF membrane (pre-activated in methanol) through wet transfer (Bio-Rad) at 75 V for 75 minutes at 4 °C. The membrane was cut horizontally according to the protein ladder (Thermo Fisher Scientific, 26619): the portion corresponding to ~55–70 kDa (or ~55–250 kDa) was used for probing FTO, whereas the ~35–55 kDa region was used for probing GAPDH. Each membrane piece was blocked with 5% non-fat dry milk in 1x PBST (PBS+0.1% Tween-20) for 1 hour at room temperature and incubated at 4 °C with the corresponding primary antibody (anti-FTO, 1:1000; anti-GAPDH, 1:1000) diluted in 5% non-fat dry milk in 1x PBST. Membranes were washed 3×10 minutes with 1xPBST at room temperature and incubated with HRP-conjugated anti-rabbit secondary antibodies (1:2000) for 1 hour at room temperature. Following three additional 3×10 minutes washes with 1xPBST, protein bands were visualized using ECL reagents (Cytiva, RPN2235) and imaged with a BIO-RAD ChemiDoc Touch Imaging system.

##### Assessment of demethylation activity of wtFTO and FTO-818 toward m^6^A and m^6,6^A in cells.

Total RNA was extracted from cells using TRIzol reagent according to the manufacturer’s instructions. Extracted RNA was treated with DNase I to remove residual genomic DNA. Then, 1.5 μg RNA was enzymatically digested into nucleosides by sequential treatment with nuclease P1 and alkaline phosphatase. Briefly, nuclease P1 in nuclease P1 reaction buffer was added and incubated at 37 °C for 30 minutes, followed by heat inactivation at 75 °C for 10 minutes. Subsequently, alkaline phosphatase in FastAP buffer was added and incubated at 37 °C for 30 minutes, followed by heat inactivation at 75 °C for 10 minutes. The resulting nucleoside mixtures were analyzed by LC-MS/MS to quantify m^6^A and m^6,6^A levels.

##### Plasmid transfection of dCas13b-FTO fusions.

To examine the global in-cell demethylation effect of the dCas-FTO fusion proteins, we transfected HEK293T cells with 1.5 μg of dCas13b-wtFTO or dCas13b-FTO-818 alone using Lipofectamine 3000 according to the manufacturer’s instructions on a 6-well plate. To perform site-specific m^6^A editing, we transfected HEK293T cells with 1.5 μg of dCas13b-wtFTO or dCas13b-FTO-818 plasmid with 1 μg of the respective gRNA plasmid using Lipofectamine 3000 on a 6-well plate. Transfected cells were incubated for 36 hours until they were harvested for downstream analysis.

##### SELECT technology for detecting m^6^A.

Detection of m^6^A at targeted sites was performed using a modified SELECT protocol^50^. For each biological replicate of the transfected cells, 2 μg of total RNA was incubated with 40 nM Up primer, 40 nM Down primer, and 5 μM dNTPs (NEB, N0446S) in 17.5 μl of 1× CutSmart buffer (NEB, B7204S). All Up and Down primers are listed in **Supplementary Table 1**. A progressive annealing program was applied: 90 °C, 80 °C, 70 °C, 60 °C, 50 °C, and 40 °C for 1 minute each, followed by 6 minutes at 40 °C. Next, 2.5 μl of enzyme mix containing 0.01 U Bst 2.0 DNA polymerase, 0.5 U SplintR ligase, and 10 nmol ATP (NEB, P0756S) was added to the annealed products for a final reaction volume of 20 μl. Reactions were incubated at 40 °C for 20 minutes, heat-inactivated at 80 °C for 20 minutes, and held at 4 °C. Subsequently, 2 μl of the final products were subjected to qPCR in a reaction containing 200 nM SELECT common primers (**Supplementary Table 1**) and 2× SYBR Green Fast qPCR Mix. The qPCR program consisted of 95 °C for 1 minute, followed by 40 cycles of 95 °C for 20 s and 60 °C for 60 s. To normalize for differences in target RNA expression level across different transfection conditions, RT-qPCR was performed using the total RNA as described above for RNA expression quantification. To calculate the relative abundance of m^6^A at a specific site after editing, we take the following steps using m^6^A1266 of *ACTB* as an example. First, we measure the Ct of the SELECT-qPCR at the m^6^A1266 site (Ct^SELECT:m6A1216^) and the Ct of *ACTB* mRNA from RT-qPCR (Ct^*ACTB*^). We then calculate ΔCt = Ct^*ACTB*^ - Ct^SELECT:m6A1266^ for on-target gRNA edited cells and NT-gRNA control cells. Next, we compute ΔΔCt = ΔCt (on-target gRNA cells) - ΔCt (NT-gRNA cells). Normalized m^6^A abundance of the edited cells by on-target RNAs is then calculated as 2^−ΔΔCt^ (Figures 6F, 6G).

## Supplementary Material

Supplementary Files

This is a list of supplementary files associated with this preprint. Click to download.

- SupplementaryFiguresandTables.docx

Supplementary Figures and Tables.
